# Reports of Societies

**Published:** 1873-12

**Authors:** Frederick Cole

**Affiliations:** Secretary; El Paso, Ill.


### Reports of Societies.

### *Woodford Co. Medical Association.*

This Association held its fall session in Edmund Burke Hall, Eureka, on Tuesday, October 21st, and was called to order at 2 o'clock by Dr. D. W. Lamme, President.

After the usual routine of business, Dr. J. S. Whitmire, from the Committee on Medical Education, made a report.

### CLINICAL EXAMINATIONS.

A large number of patients from different parts of the county had gathered, to be examined and prescribed for. This has proved to be a very interesting and profitable diversion in the exercises of the Association. Dr. J. S. Whitmire was appointed Master of Clinics, and Dr. G. F. Roberts, of Lacon, Assistant. Among the cases presented was a boy, some twelve years old, with extrophy of the bladder.

The Board of Censors presented the names of Drs. Robert G. Allen, G. Newkirk, and D. Slemmens, and on motion they were duly elected members.

Dr. J. S. Whitmire read a paper on "Criminal Abortion." And on motion the doctor was requested to have the same published in the CHICAGO MEDICAL JOURNAL.

The President, Dr. Lamme, read from his clinical notes, a detailed description of two cases of obstruction of the bowels, occurring in his practice.

Clinical reports were also made by Drs. N. B. Crawford, J. S. Whitmire, A. H. Kinnear, and G. Newkirk.

Prof. B. J. Radford, of Eureka College, was unanimously made an honorary member of the Association.

By invitation the Association voted to meet the Marshall County Medical Society in joint session, at Wenona, on the first Tuesday in January.

Prof. B. J. Radford delivered a popular lecture in the evening to a large audience, composed of the Professors, students of the college, and citizens in general, in addition to the members of the Association. Subject, "Life and its Preservation."

This Association was organized in October, 1870; and though quite small in numbers in the beginning, it has steadily grown, till now it numbers over thirty active members. Real evidence of a primary medical education from a reputable school, and a good moral and professional standing, *must* be given.

FREDERICK COLE, M.D., *Secretary.*

EL PASO, ILL., October, 1873.

